# Supplementary material for: Fate mapping reveals mixed embryonic origin and unique developmental codes of mouse forebrain septal neurons
Source: Commun Biol. 2022 Oct 27;5:1137. doi: 10.1038/s42003-022-04066-5 (PMC9613704; doi:10.1038/s42003-022-04066-5)
Supplement: Supplementary file 2 — Supplementary Information [file 42003_2022_4066_MOESM2_ESM.pdf]

Supplementary information

Fate mapping reveals mixed embryonic origin and unique developmental codes of mouse forebrain septal neurons

Lorenza Magno[1#], Zeinab Asgarian[1], Migle Apanaviciute[1], Yasmin Milner[1], Nora Bengoa-Vergniory[1], Anna Noren Rubin[1] and Nicoletta Kessaris[1#]

1 Wolfson Institute for Biomedical Research and Department of Cell and Developmental Biology, University College London, WC1E 6BT London, UK

# Corresponding authors:
Nicoletta Kessaris, Lorenza Magno
Wolfson Institute for Biomedical Research
University College London
Gower Street
London WC1E 6BT
UK
n.kessaris@ucl.ac.uk, l.magno@ucl.ac.uk

Supplementary Fig. 1

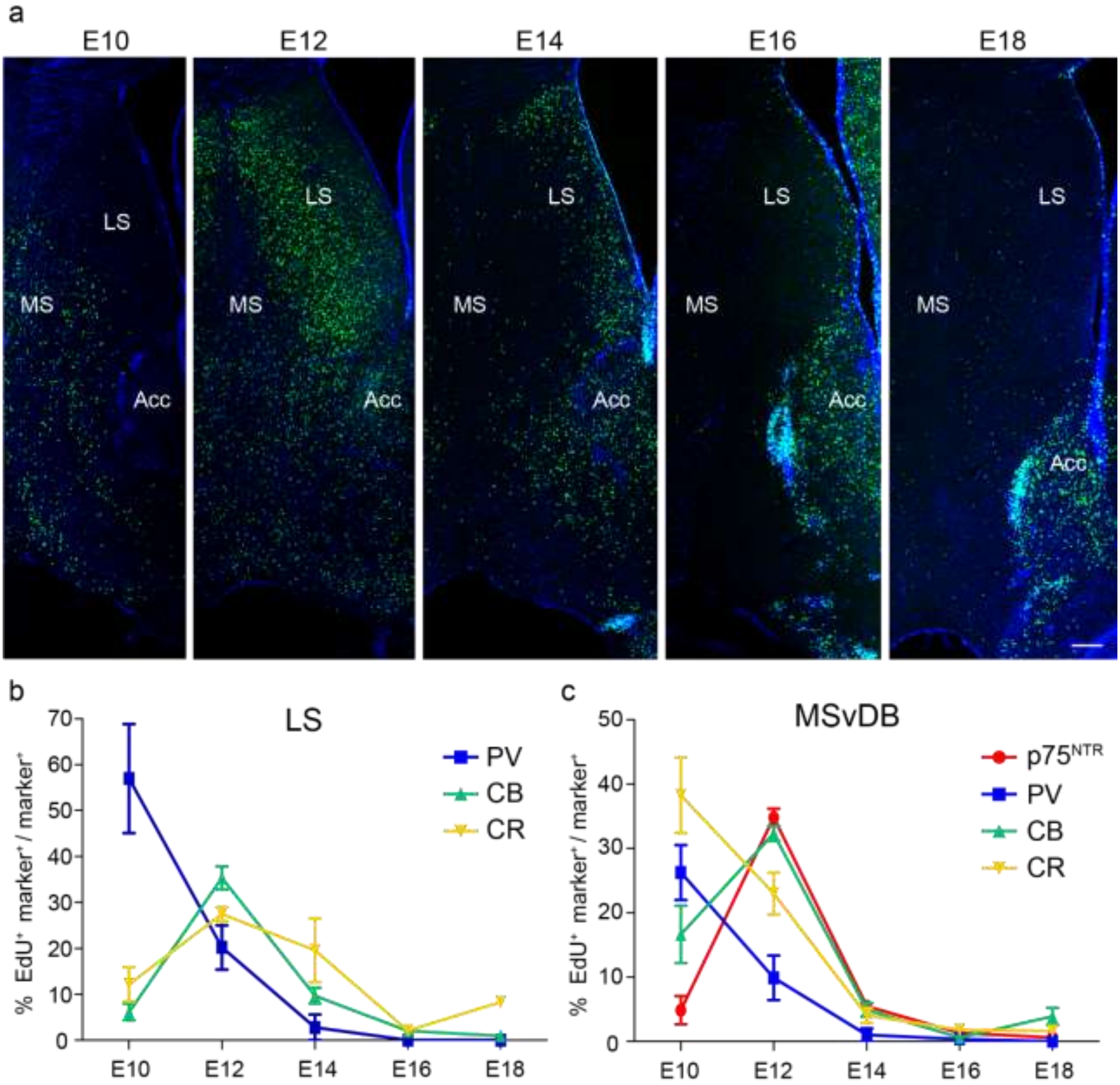

Supplementary Fig. 1: Birth-dating septal neurons

a. Representative images for EdU labelling on P30 brains after injection at different embryonic time points as indicated. LS, lateral septum; MS, medial septum; Acc, shell of the nucleus accumbens. Scale bar: 200 μm.

b. Time course of LS neuron generation. n = 3 embryos at each stage except the following where n = 2: CB E16 and CR E18.

c. Time course of MSvDB neuron generation. n = 3 embryos at each stage except CB E16 where n = 2.

All data show mean ± SEM. Source data are provided as a Supplementary Data 1 file.

Supplementary Fig. 2

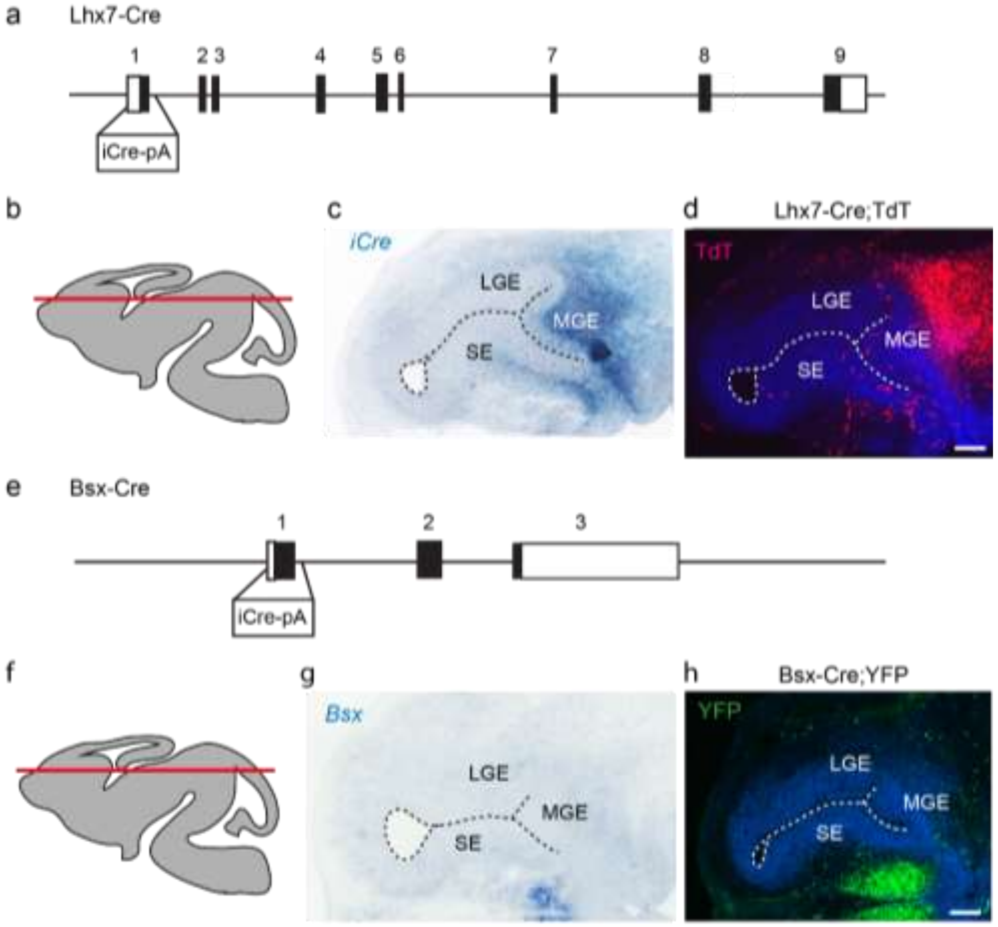

Supplementary Fig. 2: Newly-generated transgenic mouse lines used for fate-mapping the septum

a. Targeting strategy for the generation of the Lhx7-Cre BAC transgenic mouse line.

b-d. b, schematic representation of a sagittal E13.5 mouse brain and the corresponding horizontal cut. c, *In situ* hybridization for *iCre* on Lhx7-Cre E13.5 brains. d, TdT detection at corresponding horizontal level in a Lhx7-Cre;TdT brain.

e. Targeting strategy for the generation of the Bsx-Cre BAC transgenic mouse line.

f-h. f, schematic representation of a sagittal E13.5 mouse brain and the corresponding horizontal cut. g, *in situ* hybridization for endogenous *Bsx* in the forebrain. h, YFP immunolabelling in Bsx-Cre;YFP mice. Boundaries between regions are indicated by a dashed line.

SE, septum; LGE, lateral ganglionic eminence; MGE, medial ganglionic eminence.

Scale bars: 200 μm.

Supplementary Fig. 3

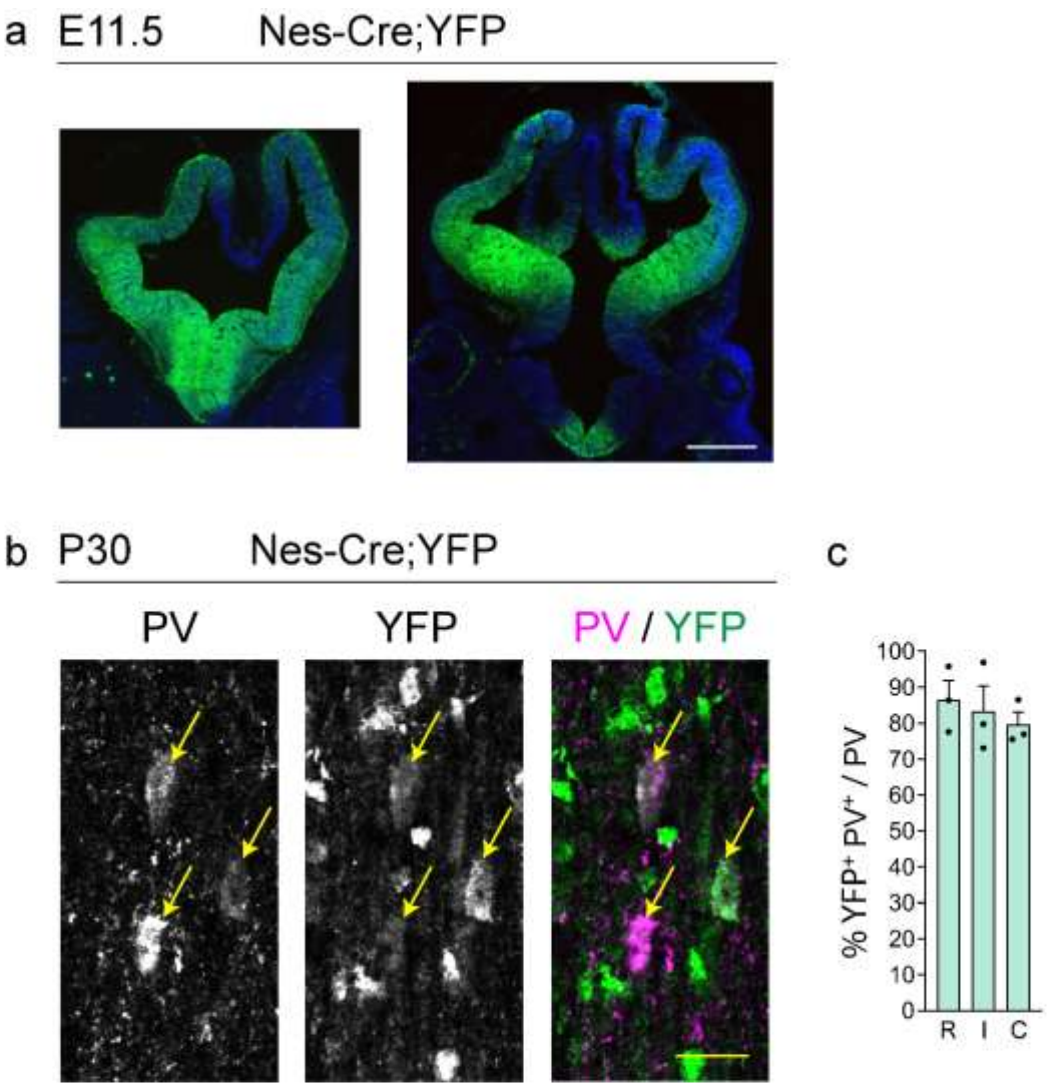

Supplementary Fig. 3: Fate-mapping MS PV neurons

a. YFP expression in forebrain germinal regions in E11.5 Nes-Cre;YFP embryonic telencephalon. YFP is detected throughout the extent of the dorsal and ventral proliferative domains.

b. Double-labeling for PV and YFP in P30 Nestin-Cre;YFP brain in the MS. White arrows indicate double labelled cells.

c. Histogram showing the percentage of neurons double labelled for YFP over the total PV population at the three rostro-caudal levels analyzed. n = 3 brains. Mean + SEM. Source data are provided as a Supplementary Data 1 file.

Scale bars: a, 200 μm; b, 20 μm.

Supplementary Fig. 4

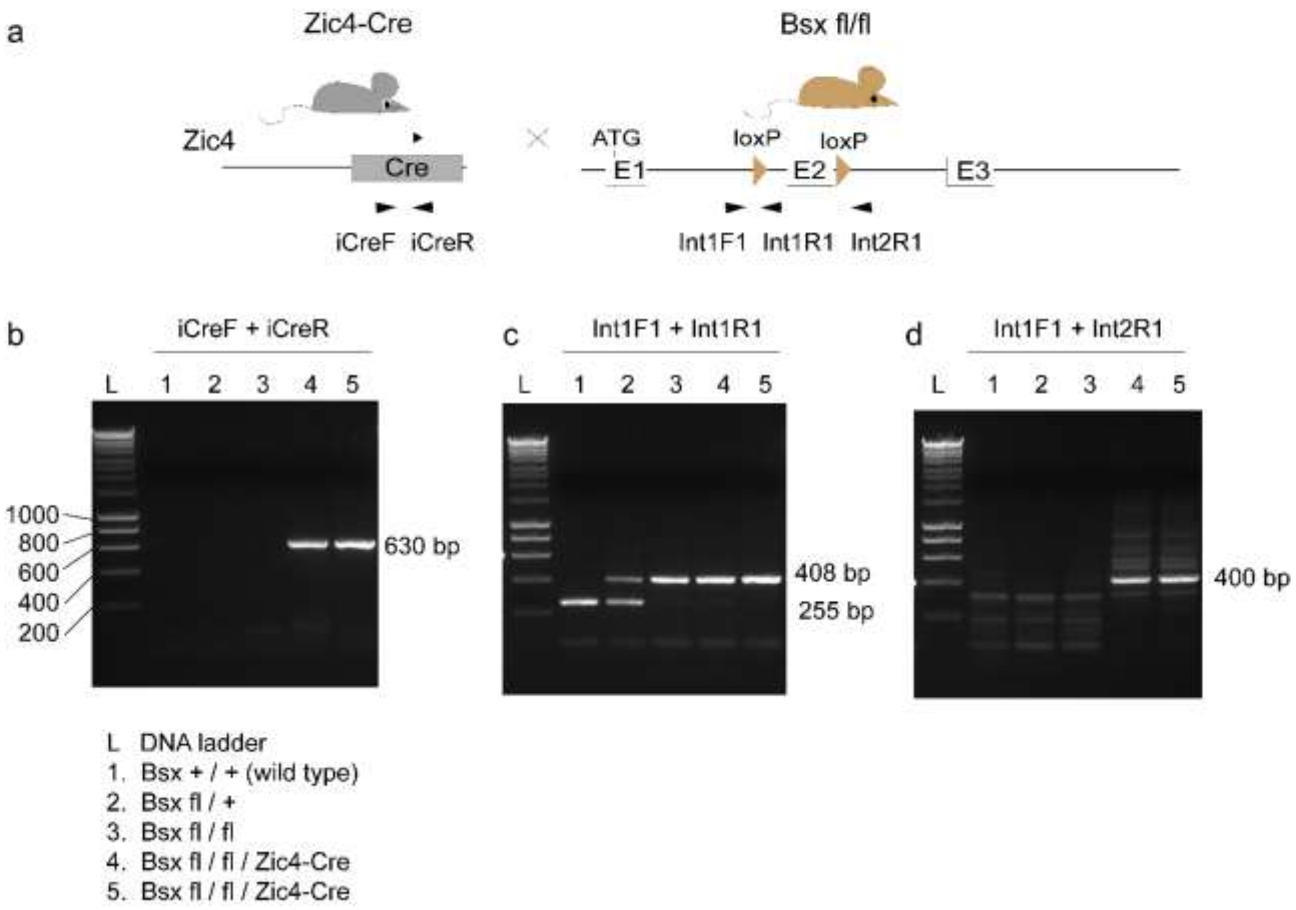

Supplementary Fig. 4: PCR detection of *Bsx* conditional alleles

a. Strategy for conditional deletion of *Bsx* in septal neurons. Approximate positions of primers used for allele detection are indicated.

b. PCR detection of iCre using primers iCreF and iCreR which amplify a band of 630 bp from genomic DNA.

c. PCR detection of the *Bsx* wild type and floxed alleles using forward (Int1F1) and reverse (Int1R1) primers in intron 1 which amplify a WT band of 255 bp or a 408 bp band corresponding to the floxed allele in genomic DNA.

d. PCR detection of the recombined *Bsx* allele using a forward primer in intron 1 (Int1F1) and a reverse primer in intron 2 (Int2R1) which amplify a band of 400 bp in genomic DNA.

Images in b-d represent unedited, uncropped images of PCR amplification reactions analyzed through agarose gel electrophoresis.

Supplementary Fig. 5

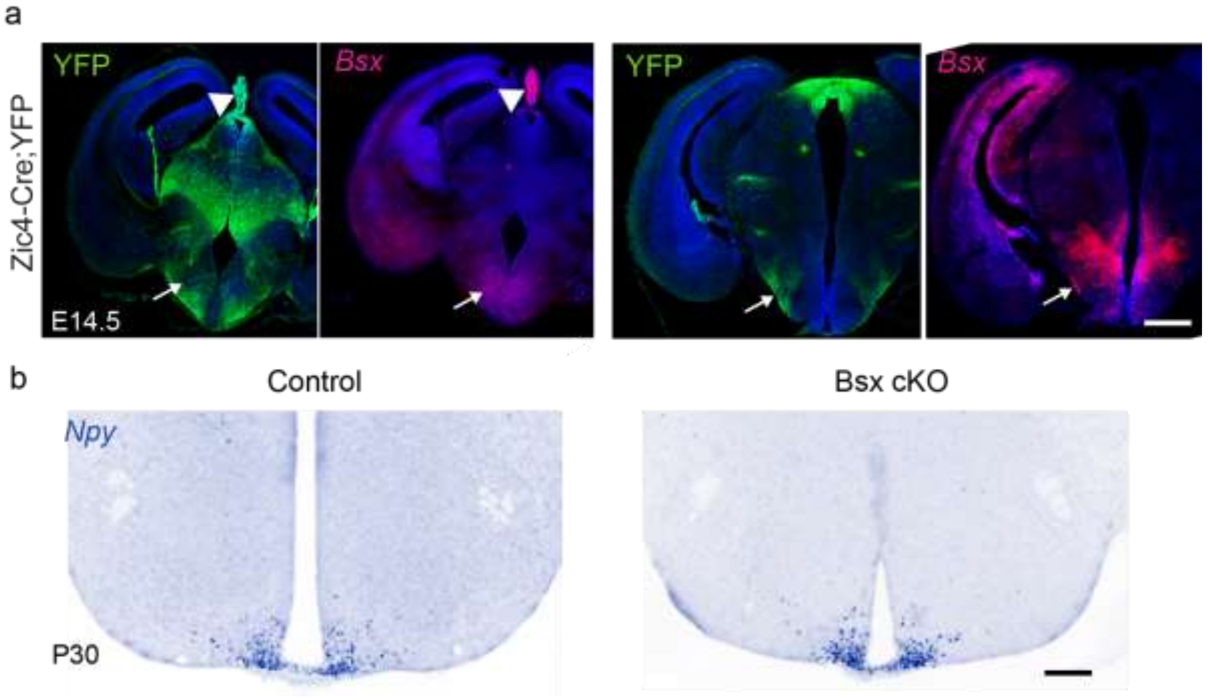

Supplementary Fig. 5: Zic4-Cre;YFP and *Bsx* expression in the hypothalamus and analysis of Zic4-Cre Bsx cKO mice

a. Serial sections of WT E14.5 Zic4-Cre;YFP caudal forebrain sections showing IHC for YFP and *in situ* hybridization for *Bsx*. Arrows indicate areas lacking co-expression in the hypothalamus, arrowheads point to co-labelling in the epiphysis.

b. *In situ* hybridization for *Npy* in the adult arcuate nucleus of control and Bsx cKO mice. An identical pattern of *Npy* expression between control and septal Bsx-cKO brains indicates absence of recombination in this region.

Scale bars: a, 200 μm; b, 100 μm.
