# Supplementary material for: Fate mapping reveals mixed embryonic origin and unique developmental codes of mouse forebrain septal neurons
Source: Commun Biol. 2022 Oct 27;5:1137. doi: 10.1038/s42003-022-04066-5 (PMC9613704; doi:10.1038/s42003-022-04066-5)
Supplement: Supplementary file 3 — Description of Additional Supplementary Files [file 42003_2022_4066_MOESM3_ESM.pdf]

Description of Additional Supplementary Files

File name: Supplementary Data 1
Description: Source data behind this paper.
