# Supplementary material for: Fate mapping reveals mixed embryonic origin and unique developmental codes of mouse forebrain septal neurons
Source: Commun Biol. 2022 Oct 27;5:1137. doi: 10.1038/s42003-022-04066-5 (PMC9613704; doi:10.1038/s42003-022-04066-5)
Supplement: Supplementary file 5 — Reporting Summary [file 42003_2022_4066_MOESM5_ESM.pdf]

nature portfolio

Corresponding author(s): NicolettaKessaris

Last updated by author(s): Sep 3, 2022

Reporting Summary

Nature Portfolio wishes to improve the reproducibility of the work that we publish. This form provides structure for consistency and transparency in reporting. For further information on Nature Portfolio policies, see our Editorial Policies and the Editorial Policy Checklist.

Statistics

For all statistical analyses, confirm that the following items are present in the figure legend, table legend, main text, or Methods section.

n/a	Confirmed	
☐	☒	The exact sample size (*n*) for each experimental group/condition, given as a discrete number and unit of measurement
☐	☒	A statement on whether measurements were taken from distinct samples or whether the same sample was measured repeatedly
☐	☒	The statistical test(s) used AND whether they are one- or two-sided<br>*Only common tests should be described solely by name; describe more complex techniques in the Methods section.*
☒	☐	A description of all covariates tested
☐	☒	A description of any assumptions or corrections, such as tests of normality and adjustment for multiple comparisons
☐	☒	A full description of the statistical parameters including central tendency (e.g. means) or other basic estimates (e.g. regression coefficient) AND variation (e.g. standard deviation) or associated estimates of uncertainty (e.g. confidence intervals)
☐	☒	For null hypothesis testing, the test statistic (e.g. *F*, *t*, *r*) with confidence intervals, effect sizes, degrees of freedom and *P* value noted<br>*Give P values as exact values whenever suitable.*
☒	☐	For Bayesian analysis, information on the choice of priors and Markov chain Monte Carlo settings
☒	☐	For hierarchical and complex designs, identification of the appropriate level for tests and full reporting of outcomes
☒	☐	Estimates of effect sizes (e.g. Cohen's *d*, Pearson's *r*), indicating how they were calculated

Our web collection on statistics for biologists contains articles on many of the points above.

Software and code

Policy information about availability of computer code

Data collection	N/A
Data analysis	N/A

For manuscripts utilizing custom algorithms or software that are central to the research but not yet described in published literature, software must be made available to editors and reviewers. We strongly encourage code deposition in a community repository (e.g. GitHub). See the Nature Portfolio guidelines for submitting code & software for further information.

Data

Policy information about availability of data

All manuscripts must include a data availability statement. This statement should provide the following information, where applicable:

- Accession codes, unique identifiers, or web links for publicly available datasets
- A description of any restrictions on data availability
- For clinical datasets or third party data, please ensure that the statement adheres to our policy

All data generated or analyzed during this study are included in this published article and its supplementary information files.

Field-specific reporting

Please select the one below that is the best fit for your research. If you are not sure, read the appropriate sections before making your selection.

☒ Life sciences ☐ Behavioural & social sciences ☐ Ecological, evolutionary & environmental sciences

For a reference copy of the document with all sections, see nature.com/documents/nr-reporting-summary-flat.pdf

Life sciences study design

All studies must disclose on these points even when the disclosure is negative.

Sample size	No statistical methods were used to predermine sample sizes. Sample sizes were chosen based on extensive experience with such experiments and following literature standards.
Data exclusions	No data were excluded from the analysis
Replication	All attempts at replication were successful
Randomization	Not relevant to this study.
Blinding	For quantification and behavioral experiments the investigators were blind to the genotypes.

Reporting for specific materials, systems and methods

We require information from authors about some types of materials, experimental systems and methods used in many studies. Here, indicate whether each material, system or method listed is relevant to your study. If you are not sure if a list item applies to your research, read the appropriate section before selecting a response.

Materials & experimental systems

n/a	Involved in the study	
☐	☒	Antibodies
☒	☐	Eukaryotic cell lines
☒	☐	Palaeontology and archaeology
☐	☒	Animals and other organisms
☒	☐	Human research participants
☒	☐	Clinical data
☒	☐	Dual use research of concern

Methods

n/a	Involved in the study	
☒	☐	ChIP-seq
☒	☐	Flow cytometry
☒	☐	MRI-based neuroimaging

Antibodies

Antibodies used	Rat anti-GFP IgG2a (Nacalai Tesque, Kyoto,Japan, #04404-84)<br>Mouse anti-calbindin (#300)<br>Rabbit anti-calbindin (#CB38a)<br>Rabbit anti-calretinin (#7697)<br>Mouse anti-calretinin (#6B3)<br>Mouse anti-parvalbumin (Chemicon Millipore #MAB1572)<br>Rabbit anti-p75NTR (Promega, Southampton, UK, #G3231)<br>Rabbit anti-TTF-1 (NKX2-1) (Santa Cruz Biotechnology, CA, #sc-13040)<br>Rabbit anti-Gsx2 (Millipore #ABN162)<br>AlexaFluor 488- conjugated, AlexaFluor 568-conjugated, and AlexaFluor 647-conjugated donkey anti-rabbit IgG or donkey anti-rat IgG or donkey anti-mouse IgG (Invitrogen, Carlsbad, CA)
Validation	All antibodies used in this study have been described previously.

Animals and other organisms

Policy information about studies involving animals; ARRIVE guidelines recommended for reporting animal research

Laboratory animals	Emx1-Cre (MGI:3761167)<br>Zic4-Cre 41 (MGI:4840322)<br>Nkx2.1-Cre (MGI:3761164)<br>Shh-Cre (JAX 005622)<br>Lhx6-Cre (JAX 026555)

Dbx1-Cre (MGI:3757955)
Nestin-Cre (JAX 003771)
Rosa26R-GFP (JAX 004077)
R26R-YFP (JAX 006148)
Rosa26R-tdTomato (JAX 007914)
Lhx7-Cre (this study)
Bsx-Cre (this study)
Bsx floxed (MMRRC:052869-UCD)

Wild animals	Wild animals were not used in this study
Field-collected samples	N/A
Ethics oversight	United Kingdom legislation (ASPA 1986)

Note that full information on the approval of the study protocol must also be provided in the manuscript.
